# The Global Redox Responding RegB/RegA Signal Transduction System Regulates the Genes Involved in Ferrous Iron and Inorganic Sulfur Compound Oxidation of the Acidophilic *Acidithiobacillus ferrooxidans*

**DOI:** 10.3389/fmicb.2017.01277

**Published:** 2017-07-12

**Authors:** Danielle Moinier, Deborah Byrne, Agnès Amouric, Violaine Bonnefoy

**Affiliations:** ^1^Laboratoire de Chimie Bactérienne, Institut de Microbiologie de la Méditerranée, Centre National de la Recherche Scientifique, Aix-Marseille Université Marseille, France; ^2^Protein Expression Facility, Institut de Microbiologie de la Méditerranée, Centre National de la Recherche Scientifique, Aix-Marseille Université Marseille, France

**Keywords:** *Acidithiobacillus ferrooxidans*, ferrous iron oxidation, inorganic sulfur compound oxidation, redox potential, two-component signal transducing system, RegB/RegA

## Abstract

The chemical attack of ore by ferric iron and/or sulfuric acid releases valuable metals. The products of these reactions are recycled by iron and sulfur oxidizing microorganisms. These acidophilic chemolithotrophic prokaryotes, among which *Acidithiobacillus ferrooxidans*, grow at the expense of the energy released from the oxidation of ferrous iron and/or inorganic sulfur compounds (ISCs). In *At. ferrooxidans*, it has been shown that the expression of the genes encoding the proteins involved in these respiratory pathways is dependent on the electron donor and that the genes involved in iron oxidation are expressed before those responsible for ISCs oxidation when both iron and sulfur are present. Since the redox potential increases during iron oxidation but remains stable during sulfur oxidation, we have put forward the hypothesis that the global redox responding two components system RegB/RegA is involved in this regulation. To understand the mechanism of this system and its role in the regulation of the aerobic respiratory pathways in *At. ferrooxidans*, the binding of different forms of RegA (DNA binding domain, wild-type, unphosphorylated and phosphorylated-like forms of RegA) on the regulatory region of different genes/operons involved in ferrous iron and ISC oxidation has been analyzed. We have shown that the four RegA forms are able to bind specifically the upstream region of these genes. Interestingly, the phosphorylation of RegA did not change its affinity for its cognate DNA. The transcriptional start site of these genes/operons has been determined. In most cases, the RegA binding site(s) was (were) located upstream from the −35 (or −24) box suggesting that RegA does not interfere with the RNA polymerase binding. Based on the results presented in this report, the role of the RegB/RegA system in the regulation of the ferrous iron and ISC oxidation pathways in *At. ferrooxidans* is discussed.

## Introduction

Among the biomining microorganisms, the strict acidophilic chemolithoautotrophic bacterium *Acidithiobacillus ferrooxidans*, that obtains its energy from the oxidation of ferrous iron [Fe(II)] and inorganic sulfur compounds (ISCs), is considered as a model. The molecular mechanisms underlying the pathways involved in aerobic Fe(II) and ISCs oxidation in *At. ferrooxidans* have been deciphered (reviewed in Quatrini et al., 2009; Bonnefoy, 2010; Bird et al., 2011; Bonnefoy and Holmes, 2012; Nitschke and Bonnefoy, 2016). The expression of the genes involved in these respiratory systems is regulated according to the available electron donor in the environment i.e., the genes involved in Fe(II) oxidation are more expressed in Fe(II)- than in sulfur-grown cells and vice-versa, those involved in ISCs oxidation are preferentially transcribed in the presence of sulfur than Fe(II) (Yarzabal et al., 2004; Quatrini et al., 2006, 2009; Bruscella et al., 2007; Sandoval Ponce et al., 2012). Furthermore, when both Fe(II) and sulfur are present in the medium, Fe(II) is immediately oxidized while sulfur oxidation takes place only after Fe(II) was completely oxidized to ferric iron [Fe(III); (Yarzabal et al., 2004; Sandoval Ponce et al., 2012)]. This observation is supported by the gene expression profile since the genes involved in Fe(II) oxidation are immediately transcribed while those involved in ISCs oxidation are expressed after Fe(II) was oxidized (Yarzabal et al., 2004; Sandoval Ponce et al., 2012). The expression of the genes involved in Fe(II) and ISCs oxidation appears therefore to depend on the oxidation state of iron [Fe(II) or Fe(III)] present in the medium.

We have proposed that a transcriptional regulator is activated when iron is present as Fe(II) and induces the expression of the genes involved in Fe(II) oxidation, while repressing those responsible of ISCs oxidation (Sandoval Ponce et al., 2012). When iron is present as Fe(III), this regulator is inactive and therefore the genes involved in Fe(II) oxidation are no longer induced, whereas those involved in ISCs oxidation are derepressed (Sandoval Ponce et al., 2012). This regulator is supposed to bind specifically to the regulatory region of the genes involved in Fe(II) and ISCs oxidation. The redox-responding global two-component signal transducing system RegB/RegA [functionally similar to PrrB/PrrA and RegS/RegR; reviewed in (Swem et al., 2001; Elsen et al., 2004; Wu and Bauer, 2008)] could be involved in this regulation since in *At. ferrooxidans*, the redox potential increases during Fe(II) oxidation to Fe(III) but remains stable during sulfur oxidation (Sandoval Ponce et al., 2012). In addition, the genes encoding a redox-sensing two-component signal transducing system belonging to the RegB/RegA family was detected in the *At. ferrooxidans* genome sequence (Quatrini et al., 2009). As shown by multiple sequence alignment, the predicted RegB/RegA proteins possess all the properties of their characterized homologs (Sandoval Ponce et al., 2012). Indeed, RegB (AFE_3136) has a transmembrane redox-sensing domain with a quinone binding site, and a transmitter cytoplasmic domain with the conserved site of autophosphorylation (the Hbox with one histidine), the redox active box with one cysteine and the ATP binding site. The response regulator RegA (AFE_3137) has a characteristic C-terminal helix-turn-helix motif DNA binding domain, as well as a N-terminal receiver domain with an “acid box” containing the aspartate residue (D68) which accepts the phosphate from the transmitter domain of the sensor RegB and a linker region between the receiver and the activation domains (Figure S1). Close to the linker region is the conserved alanine residue (A102) (Figure S1) which, when mutated to serine, locks RegA in a stable conformation that mimics the phosphorylated state of the wild type protein. RegA-A102S allows the constitutive expression of the target genes in the absence of RegB and have a similar DNA binding affinity as the phosphorylated wild type RegA (reviewed in Elsen et al., 2004; Wu and Bauer, 2008). In *At. ferrooxidans*, the *regBA* operon is not only expressed at higher levels in the presence of Fe(II) than sulfur (Amouric et al., 2009; Quatrini et al., 2009), but is also immediately transcribed in the presence of these two electron donors (Amouric et al., 2009), suggesting an autoregulation as observed for this operon in other bacteria. Finally, preliminary data support that the response regulator RegA (PrrA or RegR) binds to the regulatory region of some genes/operons involved in Fe(II) or ISCs oxidation (Amouric et al., 2009; Sandoval Ponce et al., 2012). The global redox-responding two-component regulatory system RegB/RegA could therefore be involved in the hierarchy observed in the utilization of the electron donor in *At. ferrooxidans* and could regulate the expression of the genes involved in Fe(II) and ISCs oxidation in response to the intracellular redox state.

The RegB/RegA global regulation system has been extensively studied in photosynthetic bacteria (reviewed in Swem et al., 2001; Elsen et al., 2004; Wu and Bauer, 2008). The RegA regulons of *Rhodobacter sphaeroides* (Imam et al., 2014), *Rhodobacter capsulatus* (Schindel and Bauer, 2016), and *Bradyrhizobium japonicum* (Torres et al., 2014) were characterized recently. In both cases, RegA was shown to regulate genes/operons involved mainly in energy-generating systems (e.g., photosynthesis, respiration, electron transport) and energy-utilizing systems (e.g., nitrogen and carbon fixation systems, iron homeostasis, motility).

The sensor kinase RegB detects the redox signal in the membrane through the ubiquinone pool (Swem et al., 2006; Wu and Bauer, 2010) and in the cytoplasm through a redox active cysteine located in its cytoplasmic domain (Swem et al., 2003; Wu et al., 2013). Once activated, RegB autophosphorylates and then transfers phosphate to the global response regulator RegA that binds to its target DNA.

Different effects of phosphorylation on RegA were reported (reviewed in Elsen et al., 2004; Wu and Bauer, 2008): (i) phosphorylation was shown to increase RegA DNA binding affinity/stability; (ii) both phosphorylated and unphosphorylated RegA could alter gene expression by binding to different sites (Swem et al., 2001; Ranson-Olson and Zeilstra-Ryalls, 2008; Schindel and Bauer, 2016); (iii) the activation of transcription of the target genes was enhanced but not the RegA binding to DNA. Such is the case for the *cbbI* and *cbbII* operons in *R. sphaeroides* for which RegA is required to activate transcription of the target gene by interacting with the LysR family protein CbbR, a specific transcriptional regulator of the *cbb* operons. This interaction enhances the affinity and the stability of CbbR for the *cbb* promoter DNA (Dangel and Tabita, 2009; Dangel et al., 2014). The phosphorylation of RegA leads to conformational changes in the CbbR/DNA complex that allow the recruitment of the RNA polymerase and therefore the transcriptional activation of the *cbb* operons.

Such a synergy between RegA and an activator could also be proposed in the case of the energy generating systems encoded by *cydAB* and *ccoNOPQ* in *R. capsulatus* (Swem and Bauer, 2002), *hemA* (Ranson-Olson and Zeilstra-Ryalls, 2008) and *nirK* (Laratta et al., 2002) in *R. sphaeroides* and the energy-utilizing system encoded by *nifA2* in *R. capsulatus* (Elsen et al., 2000). RegA could also function in synergy with a repressor as shown in *R. capsulatus* for the *puf* promoter involved in the synthesis of the photosystem (Gregor et al., 2007). RegA could also behave as an anti-repressor by competing with the binding of a repressor. Such is the case in the transcriptional activation of the energy-producing systems encoded by *cioAB* in *P. aeruginosa* (Comolli and Donohue, 2002) or *puc* operon in *R. capsulatus* (Bowman et al., 1999). RegA could also repress transcription by competing with the binding of an activator as shown for the energy-producing systems encoded by the *hupSLC* (Elsen et al., 2000) or *dorCDA* (Kappler et al., 2002) operons of *R. capsulatus*.

In this work, to better understand how the RegB/RegA redox-responding global two component system controls the respiratory pathways in *At. ferrooxidans*, the binding of different forms of RegA (DNA binding domain, wild-type, unphosphorylated and phosphorylated-like forms of RegA) on the promoters of the genes involved in Fe(II) and ISCs oxidation pathways have been compared by electrophoretic mobility shift assays. In addition, to determine whether RegA is a repressor or an activator of the transcription of these genes, binding of RegA relatively to the RNA polymerase binding site has been analyzed. Based on the results obtained, a model is proposed to explain the RegA mediated regulation of the Fe(II) and ISCs oxidation pathways in *At. ferrooxidans*.

## Materials and methods

### Strains, growth conditions, and plasmids

*Escherichia coli* strains and plasmids used in this study are listed in Table 1.

**Table 1:** *Escherichia coli* strains and plasmids used in this study.

**Strain or plasmid**	**Genotype or main characteristics**	**Purpose**	**Origin/References**
**STRAINS**			
TG1	*supE Δ(mcrB-hsdSM)5 thi-1* Δ(*lac*–*proAB*) F'[*traD36 proAB lacI*^q^ *lacZ*Δ*M15*]	plasmid propagation	Lucigen
HI-control 10G	*mcrA* Δ(*mrr*-*hsdRMS*-*mcrBC*) *endA1 recA1* Φ80d*lacZΔM15* Δ*lacX74 araD139* Δ(*ara,leu*)*7697 galU galK rpsL* (Str^R^) *nupG* λ^−^*tonA* /Mini-F *lacI^q1^*(Gent^R^)	plasmid propagation	Lucigen
BL21 (DE3)	[F^−^*ompT, hsdS*(*r_*B*_*^−^, *m_*B*_*^−^), *gal, dcm* λ(DE3 [*lacI lacUV5*-T7 gene *1* ind1 sam7 nin5]	RegA-HTH-6His production	Novagen
HI-control BL21(DE3)	F- *ompT hsdSB(rB- mB-) gal dcm λ*(DE3 [*lacI lacUV5*-T7 gene 1 ind1 sam7 nin5]/mini-F *lacIq1*(GentR)	6His-SUMO-RegA, 6His-SUMO-RegA_D68A and 6His-SUMO-RegA_A102S production	Lucigen
DH5α	[F^−^Φ80*lac*Z*ΔM15* Δ(*lacZYA*-*argF*) U169 *recA1 endA1 hsdR17* (rK–, mK+) *phoA supE44* λ– *thi*-*1 gyrA96 relA1*]	*regA* mutagenesis	Taylor et al., 1993
**PLASMIDS**			
pET21	ampicillin resistance; T7 transcription start; C-terminal 6His•Tag sequence	RegA-HTH-6His production	Novagen
pETite N-His Sumo kan	kanamycin resistance; T7 transcription start; N-terminal 6His•SUMO Tag sequence	6His-SUMO-RegA, 6His-SUMO-RegA_D68A and 6His-SUMO-RegA_A102S production	Lucigen
RegA-HTH-6His/pET21	DNA binding domain of RegA fused to a C-terminal 6His•Tag sequence	RegA-HTH-6His production	This study
6His-SUMO-RegA/pETite plasmid	RegA fused to a N-terminal 6His•SUMO	6His-SUMO-RegA production	This study
6His-SUMO-RegA_D68A/pETite	the conserved D68 residue of RegA was substituted for an alanine residue	6His-SUMO-RegA_D68A production	This study
6His-SUMO-RegA_A102S/pETite	the conserved A102 residue of RegA was modified to a serine residue	6His-SUMO-RegA_A102S production	This study

*E. coli* strains were grown routinely at 37°C with vigorous shaking in LB medium (Ausubel et al., 1998) with 50 μg ml^−1^ ampicillin or kanamycin. *At. ferrooxidans* type strain ATCC 23270^T^ was grown as previously described (Sandoval Ponce et al., 2012).

### DNA manipulations

*At. ferrooxidans* genomic DNA was prepared with the NucleoSpin Tissue kit (Macherey-Nagel). Go*Taq*® polymerase (Promega) was used for routine PCR and Platinum® *Taq* DNA polymerase (Life Technologies) or PrimeStar Max DNA Polymerase (Takara) for cloning purposes as recommended by the manufacturers. Primers used for PCR amplification and sizes of the expected PCR products are described in Table S1. Restriction enzyme digestions and DNA ligations were performed according to the manufacturer's recommendations (New England Biolabs). DNA fragments were analyzed by electrophoresis in agarose gel, and, when necessary, purified using Amicon® Ultra-0.5 centrifugal filter units (Millipore). Recombinant plasmids were introduced into *E. coli* TG1 (for RegA-HTH) or HI-control 10G (for RegA) competent cells as described in (Chung and Miller, 1988). Screening was performed by PCR with oligonucleotides hybridizing on both sides of the vector multiple cloning sites (pET-T7 and T7-ter for pET21, SUMO-F and T7-ter for pETite, Table S1). The recombinant plasmid was extracted with the Wizard® Plus SV DNA Purification System (Promega), checked by nucleotide sequencing (GATC Biotech) and then introduced into BL21 (DE3) (for RegA-HTH) or HI-control BL21(DE3) (for RegA) for gene expression.

### Plasmid constructions

The DNA coding for the C-terminal DNA binding domain of RegA (RegA-HTH) was amplified by PCR with RegAHTH-SacSD and RegA-XhoR primers (Table S1), digested with *Sac*I and *Xho*I and cloned into pET21 digested with *Sac*I and *Xho*I in order to get RegA-HTH-6His/pET21 plasmid encoding the recombinant protein RegA-HTH (60 amino acids) fused to a C-terminal hexahistidine tag. The DNA fragment corresponding to the RegA peptide was amplified with RegA-Sumo-For and RegA-sumo-Rev primers (Table S1). The amplified product was cloned into pETite N-His Sumo kan expression vector according to the manufacturer's instructions to get 6His-SUMO-RegA/pETite plasmid. This plasmid allows the production of RegA recombinant protein (191 amino acids) fused to a N-terminal hexahistidine-SUMO tag. SUMO is a small protein (100 amino acids) that aids the expression and solubility of proteins (Malakhov et al., 2004) while the histidine motif allows purification of the fusion protein by metal affinity chromatography.

### Mutagenesis of *regA*

The conserved D68 residue that is phosphorylated by RegB, was substituted for an alanine residue to get 6His-SUMO-RegA_D68A. The conserved A102 residue, located near the linker region between the receiver and the activation domains, was modified to a serine residue to obtain 6His-SUMO-RegA_A102S. These two mutants were constructed by site-directed mutagenesis by PCR amplification of 6His-SUMO-RegA/pETite plasmid with RegA-D68A_F and RegA-D68A_R primers to generate 6His-SUMO-RegA_D68A on the one hand, and with RegA-A102S_F and RegA-A102S_R to get 6His-SUMO-RegA_A102S on the other hand (Table S1). To get RegA-D68A, 50 ng plasmid DNA template was mixed with 0.25 μM oligonucleotides, 0.25 mM dNTPs, 2.5 units of *Pfu* Turbo high fidelity DNA polymerase (Agilent technologies) in the reaction buffer provided by the manufacturer in a 50 μl final volume. The PCR program consisted in an initial denaturation step at 95°C for 2 min, followed by 15 cycles of 30 s at 95°C, 30 s at 55°C, 6 min at 72°C. To digest the parental DNA template, ten units of *Dpn*I was added and the mixture was incubated at 37°C for 3 h. To generate RegA-A102S, PrimeStar (Takara) high fidelity DNA polymerase was used and the PCR program consisted in 15 cycles of 10 s at 98°C, 10 s at 55°C and 3 min at 72°C. *Dpn*I treatment was as described above. Mutated plasmids were introduced into *E. coli* DH5α competent cells. Screening was performed by PCR with oligonucleotides T7-ter and SUMO-F that flank the cloning site of the vector. D68A mutation was checked by *Bst*Y1 digestion of the amplified PCR fragment since the mutation will lead to the loss of this restriction site (ggatct to ggctct). The recombinant plasmids were extracted with the Wizard® Plus SV DNA Purification System (Promega), checked by nucleotide sequencing (GATC Biotech) and then introduced into HI-control BL21(DE3).

### Production and purification of His-tagged recombinant proteins

#### Expression and purification of RegA DNA binding domain (RegA-HTH-6His)

BL21 (DE3) cells carrying RegA-HTH-6His/pET21 plasmid were grown at 37°C in LB supplemented with ampicillin to an OD_600_ of 1, then induced with 0.4 mM IPTG and left at 30°C for 3 h. Cells were washed in 20 mM Tris pH 7.4, 150 mM NaCl, resuspended in 20 mM Tris pH 7.4, 500 mM NaCl, 10 mM imidazole and disrupted by passing them twice through a French press. After centrifugation (13,000 × g for 1 h at 4°C), the supernatant was loaded onto a nickel column HisTrap (GE Healthcare) according to the manufacturer's instructions. RegA-HTH-6His tagged recombinant protein was eluted with a step gradient 40, 100, 200, 350, and 500 mM imidazole. RegA-HTH was recovered in the 350 mM imidazole fraction. This fraction was washed and concentrated in 20 mM phosphate buffer pH 7.4, 150 mM NaCl and kept at 4°C.

#### Expression and purification of 6His-SUMO-RegA, 6His-SUMO-RegA_D68A and 6His-SUMO-RegA_A102S

HI-control BL21(DE3) cells carrying 6His-SUMO-RegA/pETite, 6His-SUMO-RegA_D68A/pETite or 6His-SUMO-RegA_A102S/pETite plasmid were grown at 37°C in LB supplemented with kanamycin to an OD_600_ of 0.4. They were adjusted to 17°C for 30 min and then induced with 50 μM IPTG at 17°C overnight. Cells were pelleted, washed in 40 mM Tris-HCl pH 8, resuspended in 50 mM Tris-HCl pH 8, 0.1 M KCl, 0.5 mM EDTA, 0.1% Triton X-100, 5% (V/V) glycerol, 0.1 mg ml^−1^ lysozyme, 0.1 mM phenylmethane sulfonyl fluoride (PMSF), 0.1 mg ml^−1^ DNase, 5 mM MgSO_4_, 5 mM CaCl_2_. After three freeze (−80°C)—thaw (4°C 30 min) cycles, the lysate was centrifuged at 13,000 × g for 1 h at 4°C to remove unbroken cells and cellular debris. The supernatant was filtered through a 0.22 μM Nalgene Unit and loaded onto a 1 ml Heparin column (HiTrap Heparin HP, GE Healthcare). The fractions containing the DNA binding protein RegA were eluted with 0.6 M KCl, 50 mM Tris-HCl pH 8, 0.5 mM EDTA, 0.1% Triton X-100, 5% (V/V) glycerol with the ÄKTA 10s Protein Purification System. The RegA enriched fractions were then loaded onto a 1 ml nickel column (HisTrap HP, GE Healthcare) and the His-tagged RegA recombinant proteins were eluted with a step gradient 25, 150, 500, and 1,000 mM imidazole in 50 mM Tris-HCl pH 8, 0.6 M KCl, 0.5 mM EDTA, 5% (V/V) glycerol with the ÄKTA 10s Protein Purification System. The recombinant RegA peptides eluted at 150 and 500 mM imidazole. They were concentrated with an Amicon Ultra-15 (cut off 10 kDa) centrifugal filter devices (Millipore) following the manufacturer's instructions. The purified proteins were kept in 40 mM Tris pH 8, 150 mM NaCl, 150 mM KCl, 0.5 mM EDTA, 5% (V/V) glycerol at −80°C.

#### Removal of SUMO tag by SUMO express protease

The 6His-SUMO tag was removed from 6His-SUMO-RegA, 6His-SUMO-RegA_D68A, 6His-SUMO-RegA_A102S by SUMO Express Protease provided in the Expresso™ SUMO T7 Cloning and Expression System (Lucigen), as described.

### General analytical procedure

Protein quantification was performed with the Bio-Rad protein assay according to the manufacturer's instructions. The purity of the preparations was checked by 12.5% SDS-PAGE by staining of the gels with PageBlue protein staining solution (Thermo Fisher Scientific). The recombinant protein was identified (i) by immunodetection with antibodies directed against the hexahistidine tag using a SuperSignal™ West HisProbe kit (Thermo Scientific) as described in the manufacturer's guidelines and (ii) by tandem mass spectrometry (MS/MS) after trypsin digestion of the total preparation and of the band with the expected molecular mass excised from SDS-PAGE as described in (Moinier et al., 2014), using the non-redundant NCBI *A. ferrooxidans* database. RegA-D68A and RegA-A102S were also digested with V8 protease GluC in 25 mM NH_4_HCO_3_ to identify the modified peptide.

### RegA modification

The full-length RegA (6His-SUMO-RegA) was chemically phosphorylated by acetyl phosphate for 1 h at 30°C in a reaction mix containing 25 μM 6His-SUMO-RegA, 25 mM acetyl phosphate and 20 mM MgCl_2_. The electrophoretic mobility shift assays were performed immediately after completion of 6His-SUMO-RegA modification.

### Electrophoretic mobility shift assays (EMSA)

DNA substrates for band shift assays were produced by PCR amplification with the Go*Taq*® DNA polymerase (Promega) and the oligonucleotides listed in Table S1 using one oligonucleotide 5′-Cy5 labeled (Eurogentec or Sigma-Aldrich). The reaction mix and conditions used were performed as previously described in (Sandoval Ponce et al., 2012). The Cy5 labeled DNA (2–10 ng depending on the PCR fragment) was incubated in a total volume of 10 μl with increasing concentrations of the purified recombinant 6His-SUMO-RegA, 6His-SUMO-RegA_D68A, 6His-SUMO-RegA_A102S or purified recombinant RegA-HTH as indicated in the Figures. The binding reaction contained 40 mM Tris–HCl pH 8, 100 mM KCl, 0.5 mM DTT, 0.05% Nonidet P40, 2 mM spermidine, 0.2 mM ethylene diamine tetraacetic acid (EDTA), 8% glycerol and 15 ng μl^−1^ herring sperm DNA as non-specific carrier DNA. After 30 min at room temperature, the reaction mixtures were separated by electrophoresis on a 6% native polyacrylamide gel previously preran 20 min and ran for 1–2 h more in 25 mM Tris-HCl pH 8.3, 0.19 M glycine, 1 mM EDTA, 200 μM spermidine at 30 mA at 4°C. The gel was then scanned using a 635 nm laser and a LPR filter (FLA5100, Fujifilm).

### Dynamic light scattering

To check the multimerization of the 6His-Sumo-RegA protein in the presence of DNA, dynamic light scattering has been performed with the Malvern Zetasizer Nano Series (NanoS, Malvern Instruments London, UK). The machine is equipped with a 633 nm He-Ne laser and a fixed scattering detection angle of 173°. The size of the particle was determined by its ability to scatter light. The light intensity was then translated into a hydrodynamic size calculated using the Stokes-Einstein equation. To determine the relative proportion of the different sizes in solution, the Mie theory was used to convert the intensity size distribution to mass. All reactions were performed in a final volume of 70 μL 40 mM Tris pH 8.0, 150 mM NaCl, 150 mM KCl, 0.5 mM EDTA and 0.125% Tween 20 in disposable micro-cuvettes ZEN0040. The viscosity and refractive index for the buffer were 1.0189 and 1.3319 cP, respectively. Each experiment consisted of three measurements with an average of 11 runs of 10 s each. We used the standard refractive index 1.450 for 6His-Sumo-RegA. The protein was analyzed at 0.1 mg ml^−1^ incubated 1h at room temperature with several concentrations of DNA. The particle size was determined by the distribution analysis based on Non-Negative Least Squares analysis with a regularization determined by an L–curve. The different weighted averages, intensities and volumes were used in conjunction to give a better idea of the multimerization of 6His-Sumo-RegA in the presence of DNA.

### Bioinformatic analyses

RegA alignments were performed with Clustal Omega (Sievers et al., 2011).

A nucleic pattern search tool (implemented within the Microscope platform, https://www.genoscope.cns.fr/agc/microscope; Vallenet et al., 2016) as well as the FIMO software (Grant et al., 2011; from the MEME Suite tools, http://meme-suite.org/; Bailey et al., 2009) were used to search for the RegA, PrrA and RegR motifs within the *At. ferrooxidans* ATCC 23270^T^ genome. For that purpose, the RegA, PrrA and RegR motifs (SSGNVRDNHYSNCSS, YSCGGC(5)GWCRMA and GNGRCRTTNNGNCGC, respectively) proposed in *R. capsulatus, R. sphaeroides*, and *B. japonicum* (Emmerich et al., 2000b; Eraso and Kaplan, 2009; Schindel and Bauer, 2016), were used as pattern models.

Perfect and imperfect palindromes were searched within the promoter regions under study, using the Palindrome tool (from the EMBOSS server, http://emboss.bioinformatics.nl/cgi-bin/emboss/).

Alignment of the promoter regions was performed using Kalign (http://www.ebi.ac.uk/Tools/msa/kalign/; Lassmann et al., 2009).

From the promoter regions under study, *de novo* discovery searches were first done with MEME (Bailey and Elkan, 1994) and GLAM 2 (Frith et al., 2008) tools from the MEME suite (Bailey et al., 2009). The obtained motifs were then compared to known motifs of prokaryotic transcription factors (Gupta et al., 2007), and finally searched within the ATCC 23270^T^ genome with the GLAM2SCAN tool [(Frith et al., 2008); MEME suite (Bailey et al., 2009)].

### RNA manipulations

Total RNA was prepared by using acid-phenol extraction (Aiba et al., 1981) modified according to (Osorio et al., 2013). Two DNase I treatments were performed: one with the DNAse I provided in the High Pure RNA isolation kit (Applied Biosystems) and one with the reagents from a Turbo DNA-free kit (Ambion). The lack of DNA contamination was checked by PCR with the primers RusA-F and RusA-R corresponding to an internal fragment of the *rus* gene on each RNA sample and on ATCC 23270^T^ genomic DNA as a PCR control. The absence of RNA degradation was controlled on an agarose gel.

Coupled RT-PCR experiments were performed using total RNA extracted from Fe(II)-grown cells with the Access RT-PCR System (Promega). The convergent primers used were designed from the region upstream of *regB*, from *regB* (AFE_3136) and from *regA* (AFE_3137) gene sequences of *At. ferrooxidans* ATCC 23270^T^ and are described in Table S1. Three controls were used: one without template to detect potential contaminations, one with genomic DNA as a positive control for PCR amplification and one with RNA not treated with reverse transcriptase to check for DNA contamination during RNA preparation.

The 5′ rapid amplification of cDNA ends (RACE) system (Invitrogen) was used to determine the transcriptional start site of genes/operons involved in Fe(II) or ISCs oxidation pathways following the manufacturer's guidelines and as described in (Moinier et al., 2014). This procedure amplifies nucleic acid sequences from a messenger RNA template between a defined internal site and unknown sequences at the 5′-end of the mRNA. Following amplification, 5′ RACE products are sequenced for characterization of the 5′ end of the mRNA. The primers used are listed in Table S1.

## Results

### The *regB* and *regA* genes constitute an operon

To determine whether *regB* and *regA* genes constitute an operon, RT-PCR experiments were performed on total RNA extracted from ATCC 23270^T^ strain grown on Fe(II). RT-PCR products of the expected size were generated with primers overlapping *regB* and *regA* (c and y in Figure S2, lane 1), with a primer located inside *regB* and a primer located 173 bp upstream from the translational start site of *regB* (b and z in Figure S2, lane 3), but not with a primer located 905 bp upstream from the translational start site of *regB* (a and z in Figure S2, lane 5). These results indicate that (i) *regB* and *regA* genes are cotranscribed, (ii) *regB* is the first gene of this operon and (iii) the transcriptional start site of *regB* is located upstream of 173 bp but downstream of 905 bp from *regB* translational start site, in agreement with the location we have determined previously (230 bp upstream of *regB* ATG translational start codon; Amouric et al., 2009).

### RegA-HTH and full-length RegA bind specifically to the regulatory region of several genes/operons involved in Fe(II) and inorganic sulfur compound oxidation

We have previously shown by EMSA that a preparation enriched in recombinant RegA tagged at its C-terminus with six histidines was able to bind to the regulatory region of the genes involved in Fe(II) and ISCs oxidation which expression depends on the electron donor present in the medium (Sandoval Ponce et al., 2012). However, this preparation showed signs of aggregation and tended to precipitate after further purification.

#### RegA-HTH

To confirm this specific binding of RegA, we first performed EMSA with the DNA binding domain of RegA (RegA-HTH).

**Figure 1:**
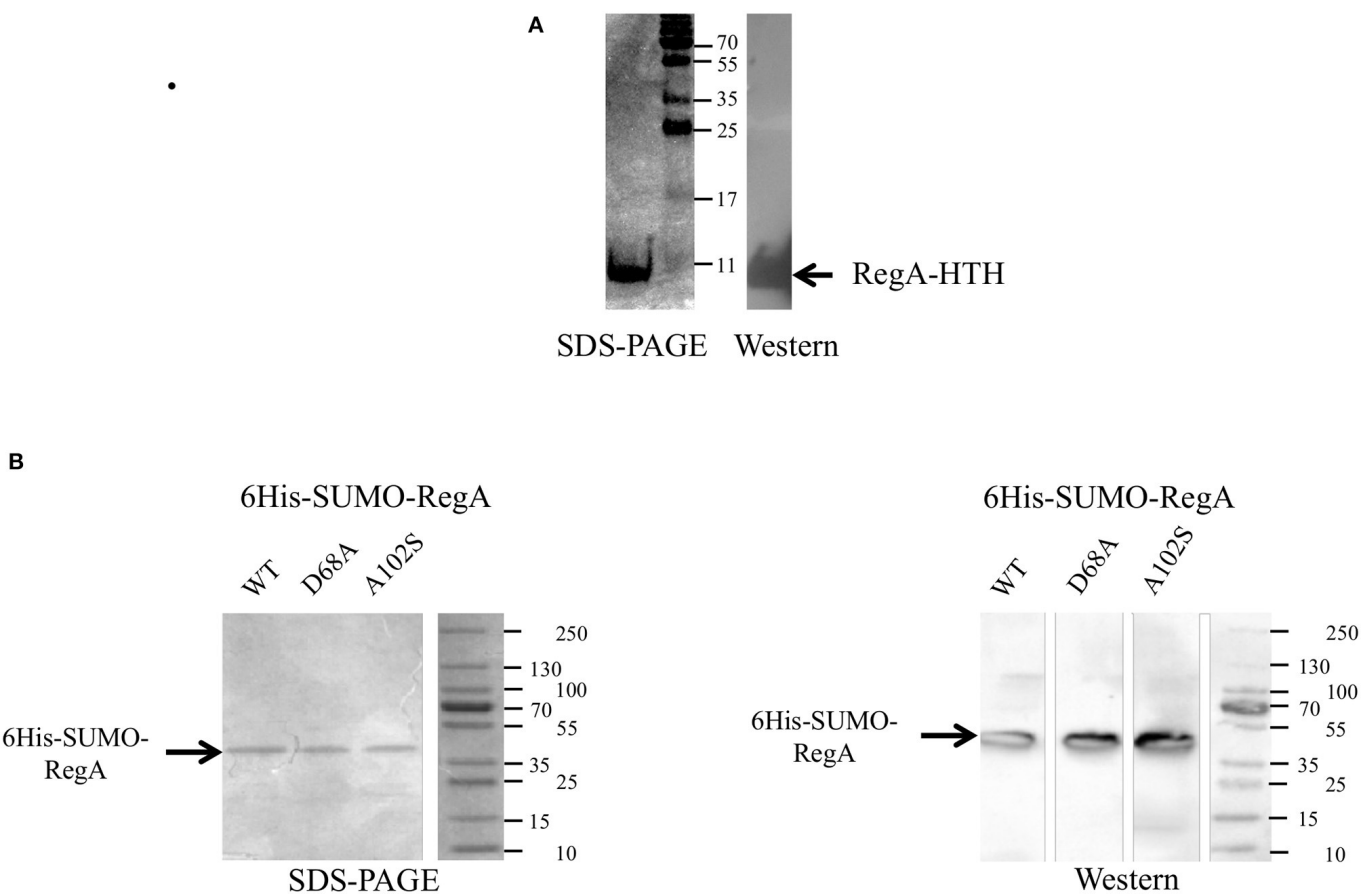
Production of the recombinant RegA-HTH-6His **(A)**, 6His-SUMO-RegA, 6His-SUMO-RegA_D68A and 6His-SUMO-RegA_A102S **(B)** in *Escherichia coli*. Coomassie brilliant blue stained SDS-PAGE and Western immunoblot with antisera raised against the hexahistidine tag. The size of the protein standards in kDa is indicated. The arrow shows the recombinant RegA-HTH-6His (7.300 kDa), 6His-SUMO-RegA, 6His-SUMO-RegA_D68A, 6His-SUMO-RegA_A102S (33.4 kDa).

The DNA binding domain of RegA (RegA-HTH) was identified by Clustal alignments with RegA homologs in which the helix-turn-helix domain has been characterized (reviewed in Elsen et al., 2004; Figure S1). The gene fragment encoding this domain (RegA-HTH) was cloned into the expression plasmid pET21 to get the recombinant protein fused to a C-terminal hexahistidine tag (RegA-HTH-6His). The identity and the purity of the recombinant RegA-HTH-6His (7.294 kDa) were checked by SDS-polyacrylamide gel (Figure 1A), immunodetection with antibodies directed against the hexahistidine tag (Figure 1A) and proteomic analysis (data not shown).

**Figure 2:**
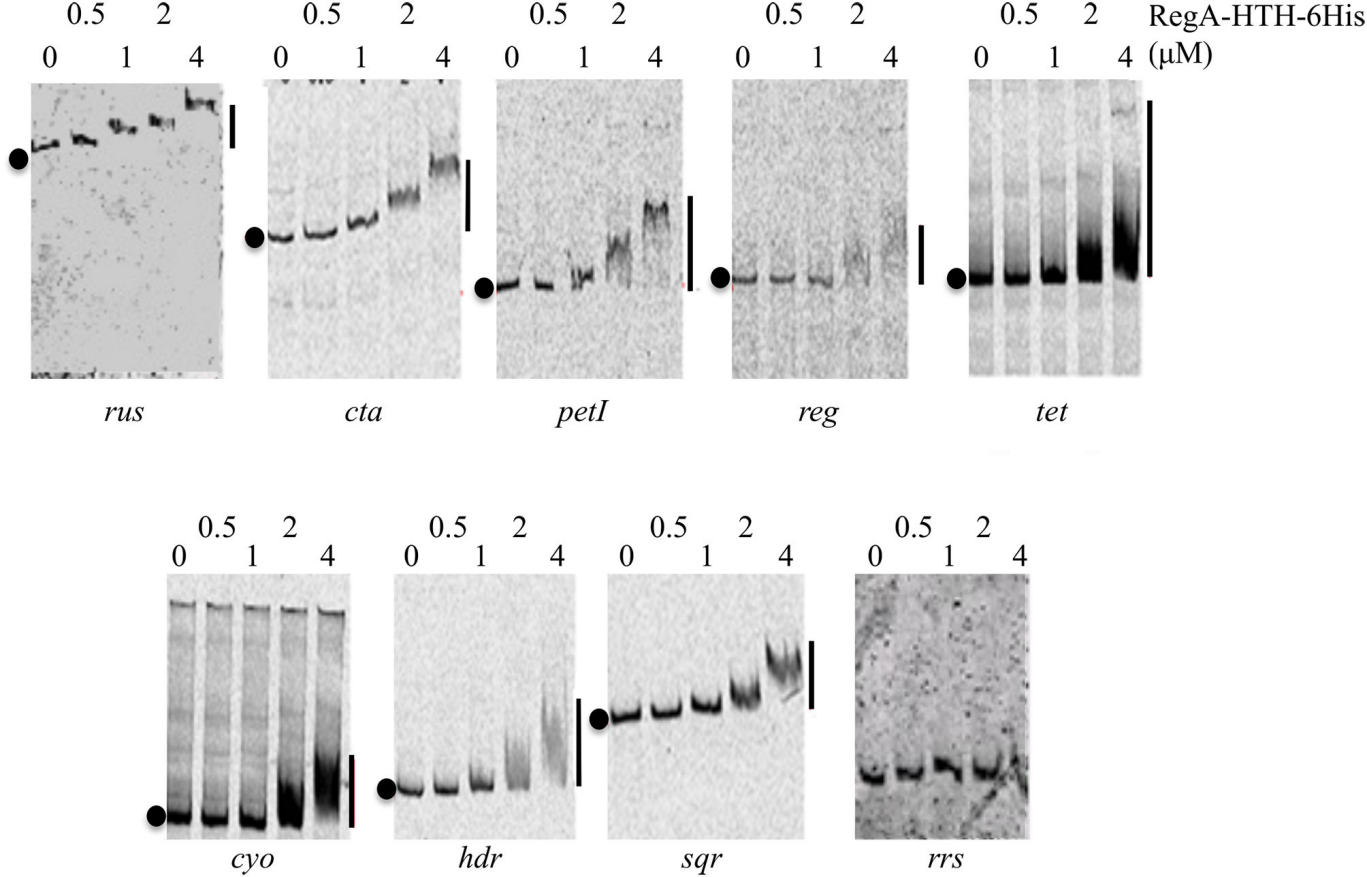
Specific binding of RegA-HTH-6His to the regulatory region of target genes involved in Fe(II) and ISCs oxidation. EMSA with the regulatory region of the *rus, cta, petI, reg, tet, cyo, hdr* operons and of *sqr* gene as well as with an internal fragment of the 16S rRNA gene from *Thiomonas arsenitoxydans* (*rrs*). RegA-HTH-6His concentrations (μM) are indicated above each lane. Black circles on the left side of the gel indicate free DNA while the line on the right, the RegA-HTH-6His complexed with DNA.

Increasing amounts of the recombinant RegA-HTH-6His were added to a reaction mixture containing the Cy5-labeled DNA fragment corresponding to the regulatory region of the *rus, cta, petI, reg* genes/operons involved in Fe(II) oxidation pathways on one hand, and *tet, cyo, hdr*, and *sqr* genes/operons involved in ISCs oxidation pathways on the other hand (Table S2). An internal fragment of the *Thiomonas arsenitoxydans* 16S rRNA encoding gene was used as negative control. No retarded band was detected with this DNA (Figure 2), even with 8 μM of RegA-HTH-6His (data not shown). On the contrary, RegA-HTH-6His /DNA complexes have been observed as one single retarded band whatever the regulator concentration and whatever the DNA probe to which RegA bound (Figure 2). Therefore, the DNA binding domain of RegA is able to bind specifically to the regulatory region of the *rus, cta, petI, reg, tet, cyo, hdr*, and *sqr* genes/operons. As observed by Dangel and Tabita (2009), the size of the RegA/DNA complexes increased with the concentration of RegA, indicating a possible multimerization of RegA on DNA. In addition, the threshold concentration of RegA-HTH necessary to observe RegA/DNA complex formation is different from one target DNA to the next (e.g., compare *rus* and *tet*), suggesting a different affinity of RegA for the target regulatory region.

#### Full-length RegA

**Figure 3:**
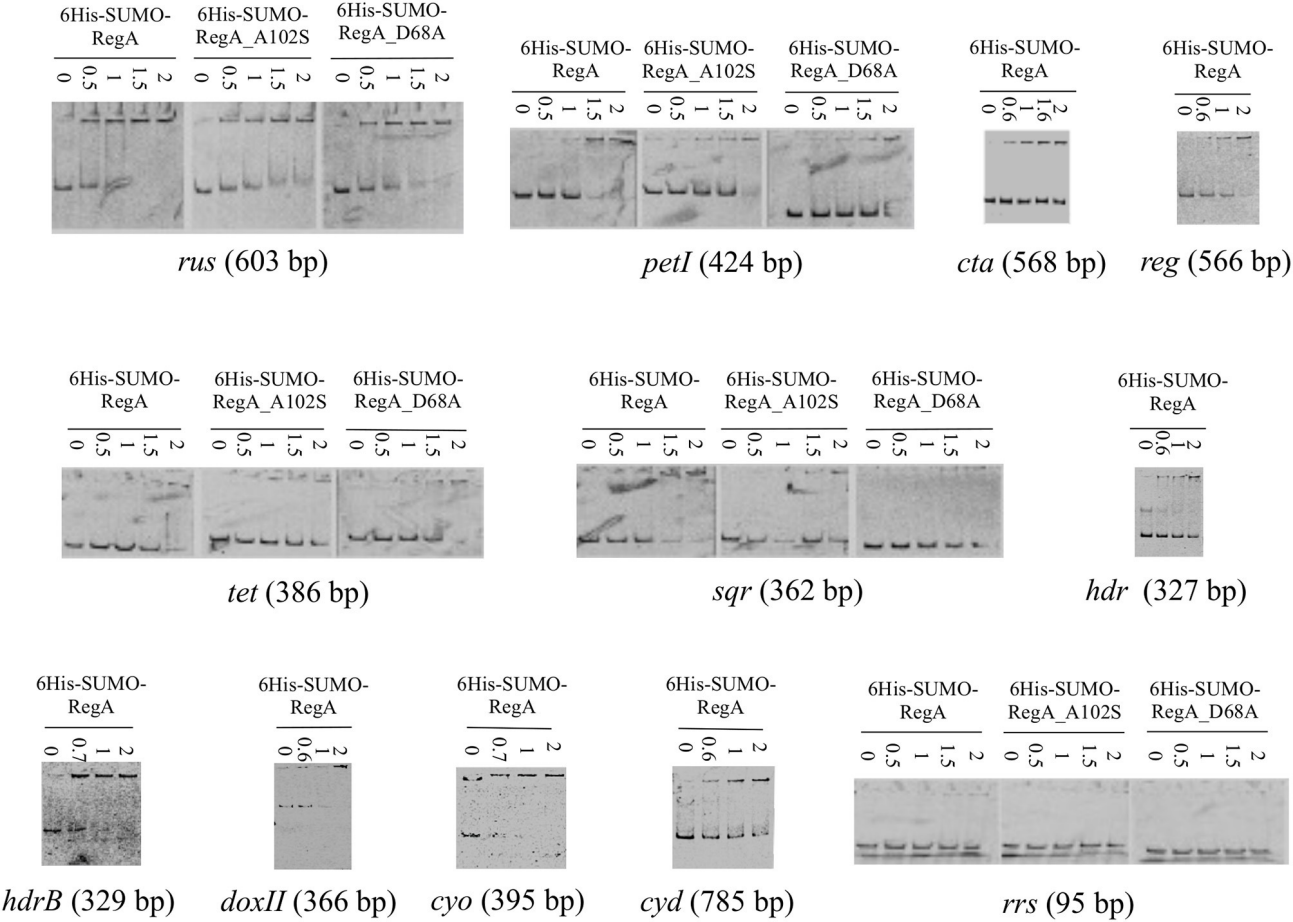
Binding of 6His-SUMO-RegA, 6His-SUMO-RegA_D68A and 6His-SUMO-RegA_A102S to the regulatory region of target genes involved in Fe(II) and ISC oxidation. EMSA with the regulatory region of the *rus, cta, petI, reg, tet, cyo, hdr, doxII*, and *cyd* operons, *hdrB* and *sqr* genes as well as with an internal fragment of the 16S rRNA gene from *Thiomonas arsenitoxydans* (*rrs*). 6His-SUMO-RegA, 6His-SUMO-RegA_D68A and 6His-SUMO-RegA_A102S concentrations (μM) are indicated above each lane.

We therefore decided to study the DNA binding capacity of the full-length RegA. For this purpose, we used the Expresso™ T7 SUMO Cloning and Expression System (Lucigen) to get the full-length RegA. The identity and the purity of the recombinant 6His-SUMO-RegA (33.382 kDa) were checked by SDS-polyacrylamide gel (Figure 1B), immunodetection with antibodies directed against the hexahistidine tag (Figure 1B) and proteomic analysis (data not shown). This preparation was used in EMSA with the same DNA fragments described above and the same results were obtained, that is 6His-SUMO-RegA is able to bind specifically to the regulatory regions of the *rus, petI, cta, reg, tet, sqr, hdr, hdrB, doxII, cyo*, and *cyd* genes/operons (Figure 3). As observed with RegA-HTH-6His, the binding of 6His-SUMO-RegA to its target DNA, led to the formation of multimeric complexes. With 6His-SUMO-RegA, these complexes were so large that they did not enter the gel. After cleavage of the 6His-SUMO tag, multimerization of RegA on DNA was also detected (data not shown). However, very quickly RegA aggregated, whichever the buffer used and the storage temperature, leading us to use the 6His-SUMO-RegA preparation in the subsequent experiments.

### 6His-SUMO-RegA multimerizes on its target DNA

**Figure 4:**
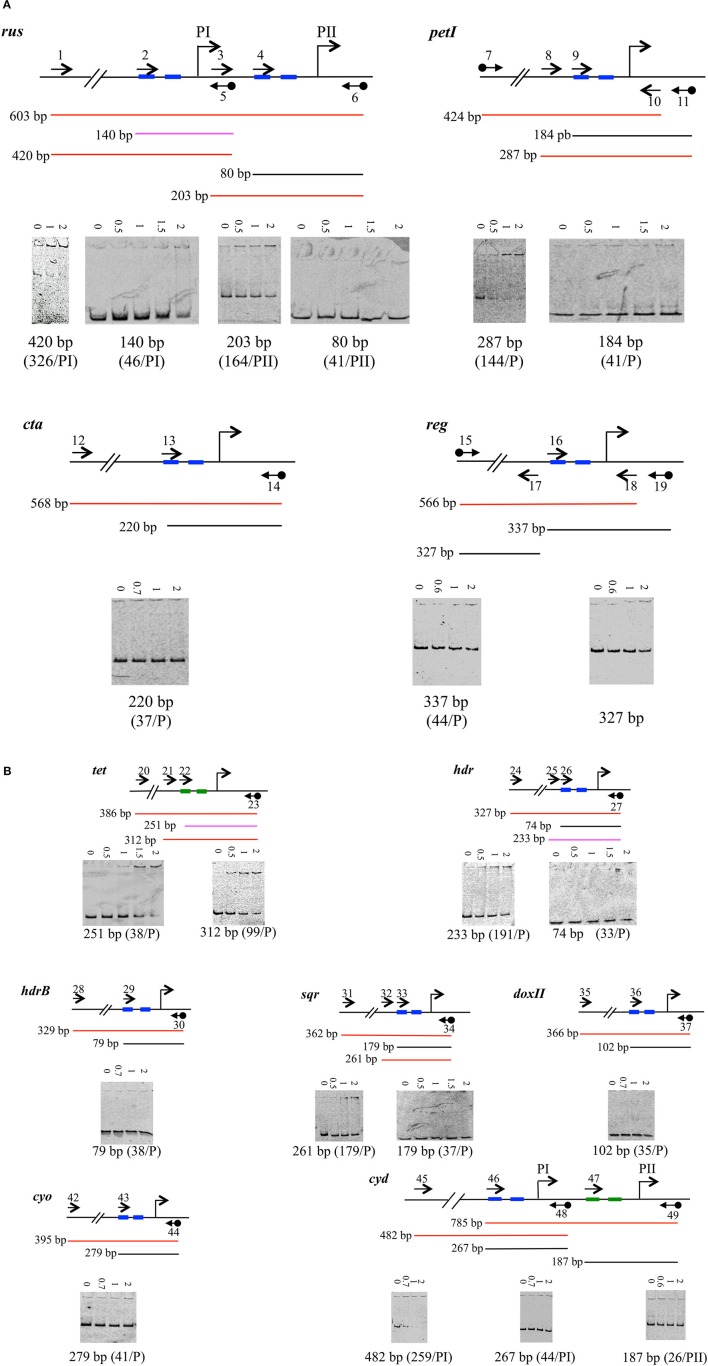
Binding of 6His-SUMO-RegA downstream to the RNA polymerase binding site of target genes involved in Fe(II) **(A)** and ISCs oxidation **(B)**. EMSA with the regulatory region of the *rus, petI, cta, reg* **(A)** *tet, hdr, hdrB, sqr, doxII, cyo*, and *cyd* **(B)** operons/ genes. For each operon/gene analyzed, a schematic representation of the DNA fragments is shown above the EMSA results. The oligonucleotides used to amplify the DNA fragments are indicated as arrows. Black diamond indicates the cyanine 5. The size of the amplicon is given. The results are represented as colors: red for positive, black for negative and pink for weak binding. 6His-SUMO-RegA concentrations (μM) are indicated above each lane. Below the EMSA is indicated the size of the amplicon. The distance from the transcriptional start site (bent arrow) is indicated in parenthesis. −35 and −10 boxes are depicted in blue, −24 and −12 boxes in green.

The multimerization of 6His-SUMO-RegA on DNA observed by EMSA was then analyzed by dynamic light scattering (DLS). 6His-SUMO-RegA is expected to have a hydrodynamic diameter of 5.24 nm for a monomer and 7.14 nm for a dimer in solution without DNA. DLS analysis showed that 6His-SUMO-RegA was polydispersed with three populations of size by intensity. To determine the relative proportion of the different sizes in solution, we used the Mie theory to convert the intensity size distribution to volume. The highest proportion of 6His-SUMO-RegA (94.6%) at 0.1 mg ml^−1^ by volume had a mean hydrodynamic diameter of 7.58 nm from three independent measurements over time (Table 2). These results suggest that 6His-SUMO-RegA in solution is a stable dimer with a melting point of 70°C. The difference between the predicted and observed size, could be that the shape of 6His-SUMO-RegA is not globular but rather elongated or elliptic. When we incubated 6His-SUMO-RegA in the presence of DNA fragments to which it was shown to bind (the 420 and 203 bp *rus* fragments; see Figure 4A), we observed an overall hydrodynamic diameter increase of approximately 20% suggesting that RegA complexed with these DNA fragments formed a multimer (Table 2), likely a tetramer. When 6His-SUMO-RegA was incubated with our negative control (16S rRNA gene; see Figure 3), there was no significant increase (Table 2). In conclusion, 6His-SUMO-RegA was shown by dynamic light scattering to be mainly dimeric in solution and to multimerize in the presence of its target DNA, in agreement with our EMSA analyses.

**Table 2:** Size distribution by volume of 6His-SUMO-RegA in the presence or absence of DNA fragments.

**Protein**	**DNA amplicon**	***N***	**Dv50**	**Inferred molecular weight (kDa)**	**Multimer**
6His-SUMO-RegA	–	3	7,58 ± 0.96	76	Dimer
6His-SUMO-RegA	*rus* 420 bp (447 fm)	3	9.22 ± 1.45	120	Tetramer
6His-SUMO-RegA	*rus* 203 bp (580 fm)	3	9.69 ± 1.75	135	Tetramer
6His-SUMO-RegA	16S 95 bp (611 fm)	1	8.10	88	Dimer

*DV50, volume based median in nm; N, number of replicates*.

### Phosphorylation of RegA does not change its DNA binding affinity for the target gene/operon regulatory region

To examine the influence of RegA phosphorylation on the efficiency of its specific binding to DNA, EMSA with phosphorylated 6His-SUMO-RegA were performed. The binding to the *rus* regulatory region of the unphosphorylated 6His-SUMO-RegA was compared to that of 6His-SUMO-RegA incubated with acetyl phosphate as phospho-donor. Both forms of RegA bound specifically to this DNA region at the same concentration (data not shown). Therefore, acetyl phosphate treatment did not change significantly the affinity of 6His-SUMO-RegA for the *rus* regulatory region. These data suggest that RegA, phosphorylated or unphosphorylated, binds to its target DNA with the same affinity. However, it is known that the efficiency of phosphorylation of response regulators with acetyl phosphate is generally <20%. Furthermore, the phosphorylated response regulators are unstable and reverse to the unphosphorylated conformation as soon as the cognate sensor no longer detects the environmental signal. Therefore, it cannot be excluded that the phosphorylation of RegA from *At. ferrooxidans* by acetyl phosphate was ineffective and/or that the phosphorylation of RegA with acetyl phosphate is particularly unstable in the conditions we have used.

To check whether unphosphorylated RegA indeed binds its target DNA, we have then mutated the *regA* gene to get (i) the inactive form 6His-SUMO-RegA_D68A of RegA that can no longer be phosphorylated by RegB (reviewed in Elsen et al., 2004; Wu and Bauer, 2008) and (ii) the phosphorylated-like form 6His-SUMO-RegA_A102S of RegA, which conformation was proposed to mimic the phosphorylated state of the wild type protein, and has been shown to activate constitutively the transcription of the target genes in the absence of RegB and to have a similar DNA binding affinity as that of the phosphorylated wild type RegA (reviewed in Elsen et al., 2004; Wu and Bauer, 2008). Both 6His-SUMO-RegA_D68A and 6His-SUMO-RegA_A102S were over-expressed and purified following the same protocol as for 6His-SUMO-RegA (Figure 1B). The specific DNA binding activities of 6His-SUMO-RegA, 6His-SUMO-RegA_D68A and 6His-SUMO-RegA_A102S to target DNA were compared in EMSA experiments. As can be seen in Figure 3, the threshold concentration of the three RegA forms necessary to observe RegA/DNA complex formation is identical for the target DNA analyzed (*rus, petI, tet, sqr*). Therefore, we concluded that the phosphorylation of *At. ferrooxidans* RegA did not modify its affinity for its target DNA.

### RegA binds generally upstream of the RNA polymerase binding site

To determine whether RegA could interfere with RNA polymerase binding to DNA, we have determined the transcriptional start site of the genes/operons involved in Fe(II) and ISCs respiratory pathways by 5′ RACE experiments and looked whether RegA binds to the region overlapping or located downstream of the RNA polymerase binding site(s) of the genes analyzed by EMSA.

5′ RACE experiments were performed on total RNA extracted from Fe(II)-grown ATCC 23270^T^ cells for *rus, cta, petI*, and *reg* operons and from S^0^-grown ATCC 23270^T^ cells for *tet, cyo, hdr, cyd*, and *doxII* operons as well as *hdrB* and *sqr* genes with the oligonucleotides depicted in Table S1. The transcriptional start site(s) of *rus* and *petI* operons in ATCC 23270^T^ are the same as the one(s) determined in *At. ferridurans* ATCC 33020^T^ (Appia-Ayme et al., 1999; Bruscella et al., 2007). Upstream of the transcriptional start site of each gene/operon obtained, a characteristic RNA polymerase binding site was detected (Table 3). Interestingly, the *tet* and the *cyd* operons have a σ54-dependent promoter, in agreement with the presence of a gene encoding a σ54-dependent transcriptional response regulator upstream of the first gene of the operons. The *cyd* operon has also a second promoter that is σ70-dependent. All the other promoters identified were σ70-dependent.

**Table 3:** Transcriptional start site(s) of the genes/operons involved in Fe(II) and ISCs respiratory pathways.

**Operon/gene**	**First gene of the operon**	**Transcriptional start site(s)**
*rus*	AFE_3153 (*cyc2*)	…GGCATGTCAGTTTTTGGGACATTTTAGTGATCGCGGCATATAATTAAAAGGCCAGATTAACATGTTT…
		…GCATAAAAAGGTGTTGCAAAGTATTACGGTTTGTACTAAATAGAACGTGTGGGTGCTGTTAGCAA…
*petI*	AFE_3107 (*cycA1*)	…TTCACAATCAAAGAGAGGGTTGACCATAATCAGTTTTTATAGTCATATATAGTCGTTATGTGGTTATAGA…
*cta*	AFE_3144 (*ctaA*)	…ATTCATGTCTTGCGGTATGCGTTATATGATGTTATGAGTATACTTGCATGCAGCGTATGAT…
*reg*	*regB* (AFE_3136)	…TGGACAGATTTGTAACTGTGTGATAACACCTGATATGATGAGGTTTCATATAGCTGGGTTAG…
*tet*	AFE_0029 (*tetH*)	…TTGTAAGAATAACAAAATAAAACAGCATCCTGTAATCATTATGCGGCTGGCACAATACATGCATAAAACATCTCGTGCTGCCGCGGGATTC…
*hdr*	AFE_2558 (*rhd*)	…GGGTTAGCCTGCCGGGCGTTTCATGTAGAATCCCCGGTCAATTTTCTTTCATGC…
*hdrB*	AFE_2586 (*hdrB*)	…AAAGCCATTTGCTTGGCAACGGAATCATTGTGGAGCATAGTGGTCAACTATTCACATGGGGGGTA…
*sqr*	AFE_1792 (*sqr*)	…AAGTTGCAACAATCTAATTTGACGAATTGGGAAGGTTCTATAATATTCCGACTAGAAATCCAATTGGGC…
*doxII*	AFE_0041	…GTAATTTTCAATTGACTGCAAAGTGTTTTATGTTTCGAGGCCGCTACAACAGATGGG…
*cyo*	AFE_0631 (*cyoA*)	…TTTGAGGCAAATCCCTATTGCTTTGCGCCCCCGCCTGCACTAGTTTTACTGCCAGGTAATCAATAGGCAG…
*cyd*	AFE_0956	…GGGTCAGCAGTTCAAGAAAGTAGACAACTTTAGCGCTATCATTACGGATAGGGTAGAGATCGATC…
		…ACAAATAAAATACAACGACCTGTTTATCATTATCCTTTCAAAGGACGCCAGGATAATAGACTATTATCAAATAATGGCACAGAGATTGCTAATACTCTGATGTCCAAATACTCGACA…

*The transcriptional start site is indicated in bold red. −35 and −10 boxes are shown in bold blue while −24 and −12 boxes are in bold green. The predicted IHF binding sites are underlined with continuous line while the Fur binding sites proposed in (Lefimil et al., 2009) is with dotted line*.

Once the RNA polymerase binding site was determined, we designed a primer overlapping the −35 box (or −24 box, in the case of a σ54-dependent promoter) for each promoter (Table S1). These primers were used to PCR amplify the region located downstream of, and including the −35 box (or −24) for each gene/operon under study. Binding of 6His-SUMO-RegA to these fragments was tested by EMSA experiments. The results are given in Figure 4. Apart from the 140 bp fragment corresponding to the PI promoter of the *rus* operon (Figure 4A) and the 251 bp fragment corresponding to the *tet* operon promoter (Figure 4B), RegA did not bind to the region overlapping the RNA polymerase binding site but rather upstream of it [see for example the 420 and 203 bp fragments of the *rus* operon (Figure 4A) and the 312 bp of the *tet* operon (Figure 4B)]. Therefore, it appears that RegA does not interfere with RNA polymerase binding to DNA, whatever the respiratory pathway involved.

### Search for the binding motif of RegA on DNA

To identify the DNA sequence recognized by RegA in *At. ferrooxidans*, the binding sites of RegA on DNA (SSGNVRDNHYSNCSS) recently determined in *R. capsulatus* (Schindel and Bauer, 2016), PrrA (YSCGGC(5)GWCRMA) in *R. sphaeroides* (Eraso and Kaplan, 2009) and RegR (GNGRCRTTNNGNCGC) in *B. japonicum* (Emmerich et al., 2000b) were searched with the nucleic pattern search implemented in the MicroScope platform and FIMO implemented in the MEME suite against the *At. ferrooxidans* ATCC 23270^T^ genome. The matched sequences were mainly detected inside of genes. In the few cases where it was found upstream, the corresponding genes were not related to energy-generating and energy-utilizing systems, in particular to none of the genes we have shown to bind RegA (data not shown). It seems therefore that RegA from *At. ferrooxidans*^*T*^ recognizes a different motif than RegA from *R. capsulatus*, PrrA from *R. sphaeroides* and RegR from *B. japonicum*.

The regions where RegA was shown to bind (Figure 4) were then analyzed for palindromes with Emboss Palindrome. Distinct perfect palindromes were identified upstream of the promoters of several but not all genes [*rus* (PI), *petI, cta, sqr, doxII, cyo, cyd* (PI and PII); data not shown]. Imperfect palindromes were detected in all the regions analyzed. However, no obvious motif present at least once in the promoter regions under study could be deduced with MEME or GLAM 2 from these imperfect palindromes.

The promoter sequences where RegA was shown to bind were aligned with Kalign. Motifs were searched with MEME and GLAM 2 in the region that aligned for all promoters and also in the entire regions where RegA was shown to bind. No motifs with a good score and low statistical significance (*E*-*value*) were detected.

In conclusion, no RegA binding motifs in the promoter regions under study can be predicted.

## Discussion

### The RegB/RegA system regulates the genes involved in Fe(II) and inorganic sulfur compound oxidation in *At. ferrooxidans*

Given that the redox potential increases during Fe(II) but not sulfur oxidation and because *At. ferrooxidans* oxidized Fe(II) before sulfur, we had put forward the assumption that the global redox responding RegB/RegA signal transduction system is involved in the regulation of the genes implied in Fe(II) and ISCs oxidation (Sandoval Ponce et al., 2012). In this paper, we have substantiated this hypothesis since the DNA binding domain of RegA (RegA-HTH; Figure 2) as well as the full length RegA (Figure 3) have been shown to bind specifically to the regulatory region of a number of genes/operons required for Fe(II) and ISCs oxidation and which are differentially expressed depending on the electron donor present in the medium (Quatrini et al., 2009). These include not only the operons/genes encoding the redox proteins allowing electron transfer from Fe(II) (*rus*) or ISCs (*cyo, cyd*) to oxygen or to NAD^+^ (*petI*), but also those encoding enzymes allowing ISCs oxidation (*sqr, doxII, hdr, tet*) and those enabling the biogenesis of the hemes present in the terminal oxidases (*cta, cyo*). Furthermore, RegA-HTH and RegA specifically recognized the regulatory region of the *regBA* operon which expression is higher in the presence of Fe(II) than Fe(III) or sulfur (Amouric et al., 2009), suggesting an autoregulation as observed for the characterized *regBA* operons (reviewed in Elsen et al., 2004; Wu and Bauer, 2008).

### RegA multimerizes on its target DNA

By electrophoretic mobility shift assays with RegA-HTH and RegA (Figures 2–4) and by dynamic light scattering of RegA (Table 2), we have shown that RegA multimerizes on its target DNA. This multimerization of RegA on its target DNA was also observed in the case of the *R. capsulatus cbbI* operon (Dangel and Tabita, 2009; Dangel et al., 2014). It could suggest either multimerization of RegA at a single site on its target DNA and/or multiple RegA binding sites on the DNA fragments used. Unfortunately, the search of putative RegA binding sites in the promoter regions under study failed. This is likely due to the degeneracy of the RegA DNA binding sequence. It is noteworthy that, while the helix-turn-helix domain of RegA in *R. capsulatus*, PrrA in *R. sphaeroides* and RegR in *B. japonicum* are 100% identical (Emmerich et al., 2000a), the DNA binding sites of RegA (SSGNVRDNHYSNCSS; Schindel and Bauer, 2016), PrrA (YSCGGC(5)GWCRMA; Eraso and Kaplan, 2009) and RegR (GNGRCRTTNNGNCGC; Emmerich et al., 2000b) are degenerated and dissimilar.

### Phosphorylation of RegA does not affect its binding to DNA

Multimerization of RegA did not require phosphorylation since multimerization was also observed with the inactive form of RegA that can no longer be phosphorylated (6His-SUMO-RegA_D68A) and with the phosphorylated-like form of RegA, which conformation was proposed to mimic the phosphorylated state of the wild type protein (6His-SUMO-RegA_ A102S; Figure 3). In addition, the phosphorylation of RegA did not affect its binding to DNA since 6His-SUMO-RegA_D68A had a similar affinity for its target DNA than 6His-SUMO-RegA_ A102S, and the wild type RegA (6His-SUMO-RegA; Figure 3). Therefore, in *At. ferrooxidans*, like in *R. capsulatus* (Dangel and Tabita, 2009), the phosphorylation of RegA did not lead to an increase of its affinity for its specific DNA target. RegA being a global regulator, we propose that RegA acts in concert with another regulator. Like in *R. capsulatus* (Dangel and Tabita, 2009; Dangel et al., 2014), phosphorylation of RegA might play a role in the formation/stability of the RNA polymerase-promoter complex.

### RegA does not compete with RNA polymerase binding to the promoter region

Having validated the hypothesis that the RegB/RegA system is controlling the genes involved in Fe(II) and ISCs oxidation, we then wondered whether RegA behaves as an activator or a repressor. Therefore, we have investigated its binding on the region corresponding to the RNA polymerase binding site. As shown in Figure 4, in most cases RegA did not bind downstream of the −35 (or −24) box, suggesting that it is not competing with the binding of the RNA polymerase. Two exceptions have been noticed: the 140 and 251 bp fragments of the regulatory regions of the *rus* and *tet* operons, respectively.

The 140 bp fragment, while overlapping the RNA polymerase binding site of PI, was just 129 bp upstream of the −35 box of the *rus* operon PII promoter. We could then consider that the RegA binding site on the 140 bp fragment concerned the transcription from PII rather than from PI. In agreement with this notion, it is mostly the PII, and not the PI, promoter which is regulated by Fe(II) (Yarzabal et al., 2004). Therefore, it can be hypothesized that RegA and the RNA polymerase do not compete for binding at PII promoter of the *rus* operon.

### Model proposed for the RegB/RegA regulation of the *At. ferrooxidans*^T^ energy pathways

Apart from the *tet* operon, we have shown that RegA was not repressing the genes/operons under study (*rus, pet, cta, reg, hdr, hdrB, sqr, doxII, cyo*, and *cyd*) by competing with RNA polymerase binding on their promoter(s). Therefore, RegA behaves either (i) as an anti-activator by competing with the binding of an activator (Elsen et al., 2000; Kappler et al., 2002), (ii) as an anti-repressor by competing with the binding of a repressor (Bowman et al., 1999; Comolli and Donohue, 2002), (iii) in synergy with a specific inducer (Elsen et al., 2000; Laratta et al., 2002; Swem and Bauer, 2002; Ranson-Olson and Zeilstra-Ryalls, 2008; Dangel and Tabita, 2009; Dangel et al., 2014) or (iv) in concert with a repressor (Gregor et al., 2007). To determine how RegA functions to control the genes/operons involved in Fe(II) and ISCs oxidation in *At. ferrooxidans*^T^, the identification of the specific regulator involved is required.

In the case of *petI* operon, the transcription factor Fur controlling iron uptake and homeostasis is likely the co-regulator of RegA since it was shown to bind also to *petI* promoter (Lefimil et al., 2009). One Fur box was predicted at the level of the −10 site of the σ70 box (Table 3), indicating that Fur inhibits RNA polymerase binding at *petI* promoter (Lefimil et al., 2009). Fur is described as repressor in the presence of high intracellular levels of Fe(II). RegA could therefore impede Fur binding on the regulatory region of the *petI* operon when Fe(II) is present.

Interestingly, a gene encoding a Rrf2 family regulator (AFE_3141) was predicted in the *cta* operon (Quatrini et al., 2009). Members of this family sense different environmental signals, in particular intracellular iron availability (Johnston et al., 2007; Hibbing and Fuqua, 2011). It is therefore tempting to propose that the Rrf2 family regulator encoded by the *cta* operon is iron responsive and regulates the clustered *cta, reg*, and *rus* operons and that Rrf2 binding is impeded by RegA in Fe(II) condition.

The *tet* operon is σ54-dependent. Therefore, its initiation requires an enhancer binding protein of the AAA-class. This activator binds usually relatively far upstream of the transcriptional start site and couples the energy produced from ATP hydrolysis to remodel the initial stable inactive conformation of the σ54-RNA polymerase bound to the −24 and −12 to a transcriptionally proficient open complex (Bush and Dixon, 2012). In the case of the *tet* operon, this enhancer binding protein is likely the σ-54 dependent transcriptional response regulator encoded by the AFE_0027 gene located upstream of *tetH* gene. In addition, the integration host factor IHF is likely required to bend the DNA to allow interaction between the σ54-dependent transcriptional response regulator and the σ54-RNA polymerase since an IHF motif is predicted upstream of the −24 box of *tet* (Table 3). Our results suggest that RegA could block the initial closed σ54-RNA polymerase-promoter DNA complex in an inefficient conformation by preventing its interaction with the response regulator leading to the inhibition of the transcription of the *tet* operon in Fe(II)-growth condition. When Fe(II) is oxidized, RegA is not synthesized, the enhancer binding protein encoded by AFE_0027 could then interact with the initial closed σ54-RNA polymerase-promoter DNA complex and activate the *tet* operon transcription when the signal is detected by its cognate sensor histidine kinase encoded by AFE_0026.

Upstream of the *cyd* operon a σ54-dependent transcriptional regulator (AFE_0957) was also predicted. However, while in the case of the *tet* operon, RegA could block the σ54-RNA polymerase in an inefficient conformation (see above and Figure 4B), this seems not to be the case for *cyd* operon (Figure 4B). We suggest that RegA could compete with the binding of either the σ54-dependent inducer (AFE_0957) or of IHF, which bends the DNA to allow interaction between the σ54-dependent transcriptional response regulator and the σ54-RNA polymerase (an IHF motif is predicted upstream of the −24 box of *cyd*, Table 3), leading to the repression of *cyd* operon transcription when Fe(II) is present.

Concerning the other genes/operons involved in ISCs oxidation (*hdr, hdrB, sqr, doxII*, and *cyo*), which are σ70-dependent, no gene encoding a transcriptional regulator could be predicted inside the operon or in their immediate proximity. The identification of the other regulator(s) of these genes/operons merits further investigation.

The results of this study strongly support the involvement of the RegB/RegA redox-responding global two-component regulatory system in the control of the genes/operons involved in the Fe(II) and ISCs oxidation pathways. Apart from the *tet* operon, RegA binding to the promoter of these genes/operons seems to not impede RNA polymerase binding. From the available data, it is tempting to propose that, in the presence of Fe(II) (i.e., low redox potential), RegA role is to block Fur or Rrf2-like repressor binding on the promoter(s) of the genes involved in Fe(II) oxidation and to prevent inducer binding on the promoter(s) of the genes involved in ISCs oxidation.

### RegB/RegA system in other acidithiobacilli

Noteworthy, the genes encoding the global redox responding RegB/RegA signal transducing system were not detected in the available genomes of *At. caldus* and *At. thiooxidans*. In addition, it has been reported that in *At. caldus*, the *tet* operon has a σ70-, and not a σ54-type promoter and is regulated by the two-component system RsrS-RsrR belonging to the EnvZ-OmpR family (Wang et al., 2016). Therefore, it seems that the regulation of the genes involved in ISC oxidation is different in iron and non-iron oxidizing acidithiobacilli. Furthermore, we have noted that, in the genome of the iron-oxidizing acidithiobacilli, *regBA* genes are always located in the same cluster than *rus* and *cta* operons, both involved in Fe(II) oxidation. This strengthens the idea that RegB/RegA are linked to the dissimilatory Fe(II) oxidation. The fact that the genetic organization of this cluster is conserved suggests that *rus, cta*, and *reg* operons were acquired together. We put forward the hypothesis that *rus, cta*, and *reg* operons are transcribed simultaneously to allow the iron-oxidizing acidithiobacilli to coordinate optimally its energy metabolism depending on the environmental conditions.

## Author contributions

VB conceived and designed the experiments. DM, DB, AA, and VB performed the experiments. DM, DB, and VB analyzed the data. DB and VB contributed to the reagents/materials/analysis tools. VB wrote the paper. All authors read and approved the final manuscript.

### Conflict of interest statement

The authors declare that the research was conducted in the absence of any commercial or financial relationships that could be construed as a potential conflict of interest.

## Abbreviations

ISCs: inorganic sulfur compounds
DLS: dynamic light scattering.
