# Supplementary material for: The Global Redox Responding RegB/RegA Signal Transduction System Regulates the Genes Involved in Ferrous Iron and Inorganic Sulfur Compound Oxidation of the Acidophilic Acidithiobacillus ferrooxidans
Source: Front Microbiol. 2017 Jul 12;8:1277. doi: 10.3389/fmicb.2017.01277 (PMC5506826; doi:10.3389/fmicb.2017.01277)
Supplement: Supplementary file 1 [file Table1.PDF]

Table S1. Primers used in this study.

Target region	Primer[$]	Primer Sequence 5' 3'*	Amplicon size (bp)[&]
RT-PCR			
rus	RusA-F	ACTGGTATGTAACTGTTGGTGCG	436
	RusA-R	GTGTATCCGAACTTGCCATCT	
regB 5' region	3197R5' (a)	CAGCCACTCACAAAACCCG	1082 with (z)
regB	3196F3' (z)	CAGGAGTATTTCTATGGCGAC	
AF23270_3122	3196/97R5' (b)	CTTGGCTTTGCTGGAGGC	351 with (z)
regB	3196R5' (c)	GATGGTCTTGGTCTTGGGC	407 with (y)
regA	3195F3' (y)	GTTGATGCTCCGAAGGGG	
Construction of the expression plasmid			
HTH domain of RegA	RegAHTH-SacSD	AAA*GAGCTC***AAGGAG**CTCCGGTATGCAAAAACGAGTTCTTGTCAGCG	182
	RegA-XhoR	TTT*CTCGAG*GCGTCGTACGGGGTGCTTAT	
RegA	RegA-Sumo-For	CGCGAACAGATTGGAGGTACCGTAAGAACTATGGAAAATAGTG	606
	RegA-sumo-Rev	GTGGCGGCCGCTCTATTAGCGTCGTACGGGGTGCTTAT	
pET21 insertion	pET-T7	GTGAGCGGATAACAATTCCCC	
	T7-Ter	GCTAGTTATTGCTCAGCGG	
pETite insertion	SUMO-F	ATTCAAGCTGATCAGACCCCTGAA	
	T7-Ter	GCTAGTTATTGCTCAGCGG	
Mutagenesis			
RegA-D68A	RegA-D68A_F	CCGGACGCCGTAGTTCTGGCTCTGCGAATGCCGGGGGTTTC	
	RegA-D68A_R	GAAACCCCCGGCATTCGCAGAGCCAGAACTACGGCGTCCGG	
RegA-A102S	RegA-A102S_F	CACCGGTTACGCCAGCATCTCAACAGCGATTGAGGCCATAAAG	
	RegA-A102S_R	CTTTATGGCCTCAATCGCTGTTGAGATGCTGGCGTAACCGGTG	
EMSA			

rrs	16S-F Cy5[#]	CACACCGCCCGTCACACC	95
	16S-rev 2	CCCCAGTCATGAAGCCTACC	
rus operon regulatory region	cyc2-R Cy5[#] (6)	GCCCCTAACCACTGGTGTTG	
	cyc2-F2-R-cy5 (5)	CATCACGATACGGATCCACT	
	cyc2-F (1)	CGTTAAGGCGACGCAGTCAG	603 with (6); 420 with (5)
	cyc2-F2 (3)	AGTGGATCCGTATCGTGATG	203 with (6)
	cyc2-F3 (2)	TGTCAGTTTTTGGGACATTTT	140 with (5)
	cyc2-F4 (4)	AAGGTGTTGCAAAGTATTACGG	80 with (6)
petI operon regulatory region	pet1-R Cy5[#] (7)	CACCGTTTTGGTTGCTTCTC	
	pet1-R2-cy5 (11)	GGCATCGCACCCATACC	
	pet1-F (10)	CCCCGAGTTAGCATATACTTATC	424 with (7)
	pet1-F2 (9)	GAGAGGGTTGACCATAATCAGT	184 with (11)
	pet1-F3 (8)	GTCGTGGGATGGTGGTC	287 with (11)
cta operon regulatory region	cta-R Cy5[#] (14)	GTAGCCCCGCCCGATACCC	
	cta-F (12)	ACCAACAAGGGATTCGGTCATAG	568 with (14)
	cta-F1 (13)	GTCTTGCGGTATGCGTTA	220 with (14)
reg operon regulatory region	RegB-R Cy5[#] (15)	GCCTGTTTGTAGATATTGTTCTGG	
	RegB-R2cy5[#] (19)	CCGATGACAGCCATTCC	
	RegB-F (18)	GCTCCACAGGGGAACGAAC	566 with (15)
	RegB-F1 (16)	TGGACAGATTTGTAACTGTG	337 with (19)
	regB-F2 (17)	TCCGACATACCTGACCCAAT	327 with (15)
tet operon regulatory region	AFE0029-R Cy5[#] (23)	GGCATATCAAGTCTCCAATC	
	AFE0029-F (20)	CAAATGAGATCGAAATACGC	386 with (23)
	AFE0029-F3 (21)	CCCCACTCCTTCCAGATA	312 with (23)
	AFE0029-F2 (22)	CATTATGCGGCTGGCACA	251 with (23)

hdr operon regulatory region	AFE_2558R Cy5# (27)	AATCCGTACATGTGCCACT	
	AFE_2558F (24)	CTTCATTACGCCCATCAG	327 with (27)
	F-rdh (26)	TAGCCTGCCGGGCGTTT	74 with (27)
	F-rdh2 (25)	GGGGGTTGGAACTTTTG	233 with (27)
hdrB regulatory region	AFE_2586Rev-Cy5# (30)	GAATACACACCGTGGAACA	
	AFE_2586For (28)	AAAGAGTACCTCCAGTTCCC	329 with (30)
	AFE_2586For1 (29)	GCTTGGCAACGGAATCA	79 with (30)
sqr regulatory region	AFE1792-R Cy5# (34)	TGCTCCTCTCGTACTTTACC	
	AFE1792-For (31)	GTCAGCGTGCAGATCATG	362 with (34)
	sqr-F (33)	AATTTGACGAATTGGGAAGG	179 with 34
	sqr-F2 (32)	CCGGTTTCTGGAGCATT	261 with (34)
doxDII operon regulatory region	AFE_0040Rev-Cy5# (37)	GCAAATCACCATTAGGAACT	
	AFE_0040For (35)	CGATGAGAACAGTGGATGA	366 with (37)
	AFE_0040F1 (36)	TTTCAATTGACTGCAAAGTG	102 with (37)
cyo operon regulatory	AFE0631-R Cy5# (44)	ATATTTCTCCCTGACGGACT	
region	AFE0631-For (42)	CACAGATGGTTTTCAGATGC	395 with (44)
	AFE_0631For1 (43)	CCCTATTGCTTTGCGCC	279 with (44)
cyd operon regulatory region	AFE_0956.R1-cy5# (49)	GATCATGAACGCCTCCAG	
	AFE_0956.R5-cy5# (48)	GCAGATTACTCCGTTCTCG	
	AFE_0956.F3 (47)	TGGCACAGAGATTGCTAATAC	187 with (49)
	AFE_0956.F2 (46)	CAGCAGTTCAAGAAAGTAGAC	785 with (49); 267 with (48)
	AFE_0956.F1 (45)	GAACAATCCACCGCTACA	482 with (48)
5' RACE			
rus	RusA-F	ACTGGTATGTAACTGTTGGTGCG	436
	RusA-R	GTGTATCCGAACTTGCCATCT	
	ainv3	ACACGTTCTATTTAATACAAACCG	

	Ainv6	GCTCATGCGCCCGGTCTTCCTGCC
	R-PIIcyc2	ACTGCTGCTAATGCTACGA
petI	ResC4-1	GGCATCGCACCCATACC
	R-petI	GCATTGGTATCCGTCGTTTT
cta	R-cta3	AGCAGGATCAGGGCAATG
	R-cta4	CGTCAGGTTATCTGCGTTT
reg	3196F	CAGGAGTATTTCTATGGCGAC
	3196Fbis	ACTCATGGTTGCTGATAGGC
tet	R-tetH1	CCATTGGAACAGCAACAG
	R-tetH2	AGCTGCGTCTCCACTTTTGT
hdr	R-rdh1	CAACTGGACAACTGGAC
	R-rdh2	CTGCACGGACATGGGAAG
hdrB	R-hdrB1	TATCCCAACCATAGAGG
	R-hdrB3	TTCGGGATTGATGCGATG
sqr	R-sqr1	CACATAGTGCCGGATTG
	R-sqr2	CCAGAACCCAGAGCTTCCTT
doxDII	AFE_0040.R3	CAGAGATAGTGGGCAACG
	AFE_0040.R1	GCAACCACGAGCTGACC
cyo	AFE0631-R-Cy5	ATATTTCTCCCTGACGGACT
	R-cyoA2	CCACCAGCAGATAAAACAGG
cyd	AFE_0956.R4	CCGCAGACTTTCCTCTCC
	AFE_0956.R1	GATCATGAACGCCTCCAG
	AFE_0956.R3	TAGGGCTGTTCATGTGCT
	AFE_0956.R5	GCAGATTACTCCGTTCTCG
	AAP	GGCCACGCGTCGACTAGTACGGGIIGGGIIGGGIIG

[$]: parenthesis refers to the name indicated in Figure 4 and Supplementary Figure S1

*: The restriction sites and the Shine-Dalgarno sequence are in italics and in bold, respectively. The mutation is underlined.
#: Cy5 stands for cyanine 5.
&: when necessary, the name of the reverse primer according to Figure 4 and Supplementary Figure S1 is given in parenthesis.
