# Supplementary material for: The Global Redox Responding RegB/RegA Signal Transduction System Regulates the Genes Involved in Ferrous Iron and Inorganic Sulfur Compound Oxidation of the Acidophilic Acidithiobacillus ferrooxidans
Source: Front Microbiol. 2017 Jul 12;8:1277. doi: 10.3389/fmicb.2017.01277 (PMC5506826; doi:10.3389/fmicb.2017.01277)
Supplement: Supplementary file 2 [file Table2.PDF]

Table S2. Genes/operons which regulatory region has been analyzed by EMSA

**Operon/gene name**	**AFE_ numbers**	**Function**	**Regulation**
rus	3153-3146	Fe(II) oxidation: electron transfer from Fe(II) to O_2	Fe(II)>S
petI	3107-3111	Fe(II) oxidation: bc_1 complex involved in the reverse electron transfer from Fe(II) to the quinone pool and to NAD	Fe(II)>S
cta	3144-3138	aa_3 cytochrome oxidase biogenesis	Fe(II)>S
regBA	3136-3137	global redox responding signal transduction system	Fe(II)>S
cyo	0631-0637	ISCs oxidation: bo_3 quinol oxidase	S>Fe(II)
cyd	0956-0953	ISCs oxidation: *bd* quinol oxidase	S>Fe(II)
hdr	2558-2550	ISCs oxidation: heterodisulfide reductase complex involved in sulfur oxidation	S>Fe(II)
hdrB	2586	ISCs oxidation: heterodisulfide reductase subunit B	S>Fe(II)
tet	0029	ISCs oxidation: tetrathionate hydrolase involved in tetrathionate hydrolysis	S>Fe(II)
sqr	1792	ISCs oxidation: sulfide quinone oxidase involved in sulfide oxidation	S>Fe(II)
doxII	0041-0046	ISCs oxidation: thiosulfate-quinone-oxidoreductase involved in thiosulfate oxidation	S>Fe(II)
