# Supplementary material for: The Global Redox Responding RegB/RegA Signal Transduction System Regulates the Genes Involved in Ferrous Iron and Inorganic Sulfur Compound Oxidation of the Acidophilic Acidithiobacillus ferrooxidans
Source: Front Microbiol. 2017 Jul 12;8:1277. doi: 10.3389/fmicb.2017.01277 (PMC5506826; doi:10.3389/fmicb.2017.01277)
Supplement: Supplementary file 3 [file Image1.PDF]

```
RegA_A.ferrooxidans MTVRTMENSADITATPVSNIMVVDDDVTFCRVLSNAFSRRGFEVCTVHDSADIIAVAELY
RegR_B.japonicum    -----MNAIAELNEQTDRSLLIVEDDKPFLERLSRAMETRGFAVTSCDTVSDGLAQIGKA
REGA_R.sphaeroides  -----MAEDLVFELGADRSLLLVDDDEPFLKRLAKAMEKRGFVLETAQSVAEGKAIAQAR
REGA_R.capsulatus   -----MAEEEFAELGSDRSLLLVDDDNAFLTRLARAMEKRGFQTEIAETVSAGKAIVQNR
                         *            .:::*:**  *   *:.*:. ***     .  :   *

RegA_A.ferrooxidans VPDAVVLDLRMPGVSGLEMISPLRSINQDIRILVLTGYASIATAIEAIKLGAVYYLTKPA
RegR_B.japonicum    APAFAVVDLRLGDGNGLDVVSALKKKRPDARAIVLTGYGNIATAVTAVKMGAIDYLSKPA
REGA_R.sphaeroides  PPAYAVVDLRLEDGNGLDVVEVLRERRPDCRIVVLTGYGAIATAVAAVKIGATDYLSKPA
REGA_R.capsulatus   APAYAVIDLRLEDGNGLEVVEALRERRPEARIVVLTGYGAIATAVAAVKMGATDYLSKPA
                     *  .*:***: . .**:::. *:. . : * :*****. ****: *:*:**  **:***

                                    Hinge           α6             α7        α8
                                     ____     _________________  _________  _____
RegA_A.ferrooxidans DADEIIARLHEKDGNPTAPVKNEFLSARRVEWEHINKVLMECNGNISAAARRLGMHRRSL
RegR_B.japonicum    DADDVVAALLSTSAE-KSELPTNPMSADRVRWEHIQRIYEMCNRNVSETARRLNMHRRTL
REGA_R.sphaeroides  DANEVTHALLAKGES-LPPPPENPMSADRVRWEHIQRIYEMCDRNVSETARRLNMHRRTL
REGA_R.capsulatus   DANDITNALLAKGEA-LPPPPENPMSADRVRWEHIQRVYELCDRNVSETARRLNMHRRTL
                    **:::   *  ..         : :** **.****:::   *: *:* :****.****:*

                    ____
RegA_A.ferrooxidans QRKMNKHPVRR
RegR_B.japonicum    QRILAKRAPR-
REGA_R.sphaeroides  QRILAKRSPR-
REGA_R.capsulatus   QRILAKRSPR-
                    ** : *:  *
```

Figure S1. Alignment of RegA from *At. ferrooxidans*^{T} with RegA homologues in which DNA binding domain has been characterized . The phosphate-accepting aspartate residue is shown in bold red. The alanine residue which, when mutated to serine, confers to RegA a stable conformation that mimics the phosphorylated state of the wild type protein, is in blue and underlined. The linker region connecting the receiver and the DNA binding domains is labeled Hinge. The helix-turn-helix domain is denoted by α6, α7 and α8. The RegA HTH domain of *A. ferrooxidans* that has been studied in this paper is denoted in italics. Identical (*), strongly similar (:), and weakly similar (.) residues are indicated below the alignment.
