# Supplementary material for: The Global Redox Responding RegB/RegA Signal Transduction System Regulates the Genes Involved in Ferrous Iron and Inorganic Sulfur Compound Oxidation of the Acidophilic Acidithiobacillus ferrooxidans
Source: Front Microbiol. 2017 Jul 12;8:1277. doi: 10.3389/fmicb.2017.01277 (PMC5506826; doi:10.3389/fmicb.2017.01277)
Supplement: Supplementary file 4 [file Image2.PDF]

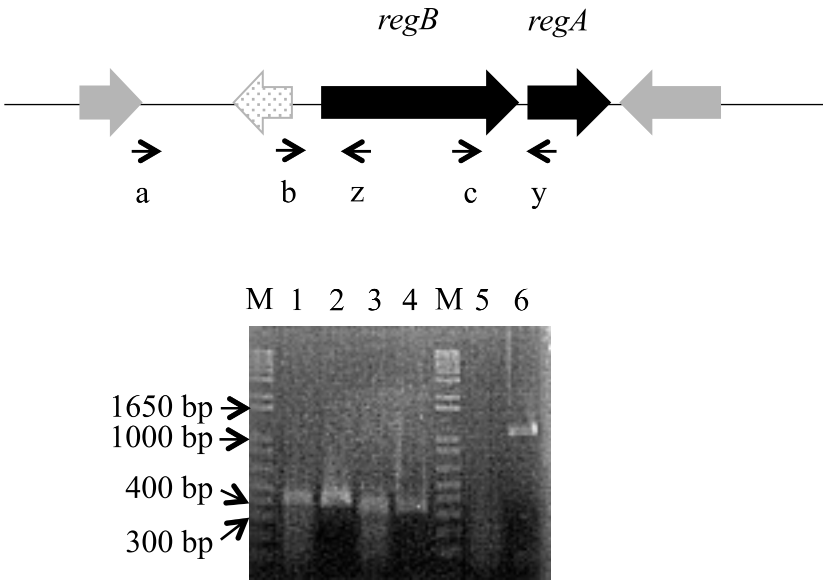

Figure S2. Genetic organization of *regA* and *regB* genes. RT-PCR amplification of the intergenic regions of the *regBA* locus between primers c and y (1, 2), b and z (3 and 4) and a and z (5,6) with total RNA (1, 3, 5) and genomic DNA (2, 4, 6) extracted from Fe(II)-grown cells. The locations of the primers used for RT-PCR experiments are shown above the map of the *regBA* cluster and listed in Supplementary Table S1. The stippling arrow represents a gene encoding a non-conserved hypothetical protein. M: 1 kb plus DNA ladder from Invitrogen.
